# Vinegar amount in the process affected the components of vinegar-baked Radix Bupleuri and its hepatoprotective effect

**DOI:** 10.1186/s12906-016-1333-4

**Published:** 2016-09-06

**Authors:** Ya Zhao, Yin-Jie Wang, Rui-Zhi Zhao, Fei-Jun Xiang

**Affiliations:** 1 Second Affiliated Hospital, Guangzhou University of Chinese Medicine, Neihuan Xilu, Guangzhou Daxuecheng, Guangzhou, 510006 China; 2 Yipinhong Pharmaceutical Co. Ltd, 17rd floor, Xinghui International Building, New city Zhujiang, Guangzhou, 510623 China; 3 The postdoctoral research station, Guangzhou University of Chinese Medicine, Neihuan Xilu, Guangzhou Daxuecheng, Guangzhou, 510006 China

**Keywords:** Vinegar amount, Vinegar-baked Radix Bupleuri, Hepatoprotective effect, Saikosaponin

## Abstract

**Background:**

Bupleuri Radix (in Chinese Chaihu), the dried roots of *Bupleurum Chinense* DC, is a traditional Chinese medicine widely used to treat fever, hepatitis, jaundice, nephritis, dizziness. When baked with vinegar, its effect is more focused on liver related disease. This paper was undertaken to determine the best vinegar amount in the processing and explore its key efficacy components.

**Methods:**

Hepatoprotective effects of Radix Bupleuri after processing with different amount of vinegar (1:5, 2:5, 3:5) were investigated on liver hurt rats, and the change of constituents were analyzed by ultra-performance liquid chromatography tandem mass spectrometry (UPLC-MS/MS).

**Results:**

With the increasing amount of vinegar, the hepatoprotective effects of vinegar-baked Radix Bupleuri (VBRB) and the content of saikosaponin b_2 _increased.

**Conclusion:**

These results suggested that vinegar amount in the process affected the pharmacological effect of VBRB significantly and saikosaponin b_2_ may be the key efficacy component of it.

## Background

Radix Bupleuri (Chaihu), the radix of *Bupleurum Chinense* DC is a well-known traditional Chinese medicine (TCM) usually used for curing influenza, fever, malaria, hepatitis, jaundice, nephritis, dizziness, bitter taste in mouth, lung diseases, cancer, and menstrual disorders in Asian countries [1–7]. Radix Bupleuri always used in liver -related disease in clinic, and modern pharmacology research also proved that radix Bupleuri had hepatoprotective effects. Xie DH et al. adopted dimethylnitrosamine –induced chronic liver fibrosis in rats, and compared protective liver action of north Chaihu and spring Chaihu on blood biochemical indexes, the content of hepatic tissue collagen protein and liver cell apoptosis. Spring Chaihu and north Chaihu have protective effect on anti- liver fibrosis in rats, north Chaihu is better than spring Chaihu on protective liver action [8]. Guinea MC showed that both the saponin fraction and the isolated compound of Bupleuri showed substantial hepatoprotective effects against *d*-galactosamine [9]. Yen MH et al. demonstrated that the hepatoprotective effects of water extract, both polysaccharide- and saponin-enriched fractions of B. kaoi in rats [10]. Mohamed L. et al. concluded that the high concentrations of triterpene saponins and polyphenolics significantly contribute to the observed hepatoprotective effects of Bupleurum spp. through reviewing the literature [11].

Prior to its usage in clinics, Radix Bupleuri is usually subjected to traditional Chinese processing techniques (PaoZhi). When Radix Bupleuri is baked with vinegar, it’s effect even more focus on liver [12–14]. Some people compared the pharmacological effects and found that after the vinegar baked process, acesodyne and bile secretion enhancing effect of Radix Bupleuri increased significantly [15, 16]. Li ZY et al. compared the chemical compositions by ^1^H NMR spectroscopy coupled with multivariate analysis and biological effects against CCl_4_ induced liver injury between raw and two processed Radix Bupleuri by different types of vinegars and found that VBRB had a more strong hepatoprotective effect, meanwhile the content of saikosaponin a and d decreased and saikosaponin b_2_ increased, and VBRB with a higher saikosaponin b_2_ seems had a better effect [17]. Saikosaponin b_2_ is a metabolite of saikosaponin d, and above results indicated that its content may be related with vinegar amount in the processing.

Ancient literature records three different amount used in the vinegar process, that is vinegar: Radix Bupleuri (1:5, 2:5, 3:5). In Pharmacopeia of China, 1:5 ratio is used, but no evidence showed which amount is the best and which component responds for the pharmacological change. Therefore, in this study, the hepatoprotective effects of VBRB with different amount of vinegar in the angry model induced by tail clamping methods was compared, and meanwhile the components of VBRB was analyzed. These data gave some clues for the processing quality control and active integrants of VBRB.

## Methods

### Plant material and processing technology with different amount of vinegar

The decoction pieces of Radix Bupleuri were purchased from Kangmei Medical Company (Guangzhou, China) and the batch number was 14061371. The purchased samples were identified by Ruizhi Zhao (the corresponding author) and voucher specimens were deposited in Materia Medica Preparation Lab of Second Affiliated Hospital, Guangzhou University of Chinese Medicine. The decoction pieces of Radix Bupleuri were soaked with vinegar (the ratio were 5:1, 5:2, 5:3, respectively) for 6 h until the vinegar was completely absorbed, then baked in electric oven at 100 °C for 2.5 h and stirred every 30 min during the time. The VBRB were taken out and cooled to room temperature for next use.

### Chemicals and reagents

Rice vinegar was purchased from Beijing Er Shang Longhe Food Co. Ltd (Beijing, China). Saikosaponin a, b_2_, c, d (all purity ≥98.0 %) was purchased from Shanghai Winherb Medical S&T Development Co. Ltd (Shanghai, China). The HPLC-grade reagents acetonitrile, and formic acid were obtained from Fisher Scientific ((Fairlawn, NJ, USA). All other chemicals were of analytical grade. Aspertate aminotransferase (AST), Alanine aminotransferase (ALT), Total bilirubin (TBIL), direct bilirubin (DBIL) and total bile acid (TBA) assay kits were purchased from Rocher Diagnostics Ltd (Germany).

### Animals and their care

SD rats, male, 280–320 g, were provided by Guangdong Medical Laboratory Animal Center, and their certificate number was 0116066. They were acclimatized in an air conditioned room at 22 ± 2 °C for 3 days with a 12 h light and 12 h darkness cycle. All animals were free to standard laboratory chow and tap water before experiment. The studies were approved by the Animal Ethics Committee of Guangdong Province Hospital of Traditional Chinese Medicine.

### Extract of VBRB for animal experiment

One-hundred grams of VBRB (1:5, 2:5, 3:5) were soaked in 10 times of water for 0.5 h, then boiled for 1 h and filtered. The residues were extracted for other two times with 8 times of water for 40 min and filtered. The filtrate was combined and condensed to 100 mL (water extract) at 60 °C under reduced pressure, and finally freeze-dried.

### Induction of liver hurt model and drug treatment

The rats were randomly divided into five groups with 10 rats each, including a normal control group, model group, VBRB processed with different amount of vinegar (1:5, 2:5, 3:5) group. Except for rats in normal control group, the tails of all other rats were clamped about 30 min by folder 4 times per day for 7 days to induce liver hurt model. Then all rats were fasted except for water for 12 h, 600 mg/kg of VBRB (1:5, 2:5, 3:5) were given to the rats in the treating group respectively. Normal control group and model group received the same amount of distilled water.

### Biochemical assays

At the end of this experiment, rats were injected 10 % chloral hydrate intraperitoneally at a concentration of 0.30 mL/100 g, and blood samples were collected from the retinal vein plexus. Blood was allowed to clot and centrifuged at 3500 rpm for 10 min, then the serum were collected for determine the content of AST, ALT, ALP, TBIL, DBIL and TBA by kits according to the manufacturers’ protocol.

### Preparation of standard solutions and VBRB samples for UPLC-MS/MS analysis

Saikosaponin a, b_2_, c, d were accurately weighed and dissolved in 70 % methanol to get standards stock solutions (0.32 mg/mL, 0.20 mg/mL, 0.18 mg/mL, 0.22 mg/mL respectively), then they were kept at 4 °C until use.

The water extract of Radix Bupleuri and VBRB with the herb concentration of 1 g/mL was obtained by using the method described in samples for animal experiment. Then the extraction was added appropriate amount of 95 % ethanol to adjust the ethanol concentration to 80 %; and the mixture was kept overnight and then centrifuged at 3500 rpm for 30 min. The residue was freeze-dried (BC1), and the supernatant was condensed by removing ethanol and further extracted by n-butanol. The n-butanol layer (BC2) and water layer (BC3) were separated and condensed, and the residues were freeze-dried, and weighted. For saikosaponins determination, dried powder of water extract were dissolved and diluted in 70 % methanol at a concentration of 0.1 mg · mL^−1^ (calculated on the amount of raw materials), then centrifuged at 3500 rpm for 10 min. The supernatant were filtered through a 0.22 μm membrane filter before injecting into UPLC-MS/MS.

### UPLC-MS/MS system

Chromatographic separation was performed on a Waters Acquity ultra performance liquid chromatography (UPLC) consisted of a binary pump and a sample manager. The MS analysis was performed using Waters Xevo TQ-S Mass Spectrometer coupled with ESI source. The analytes were separated on a Thermo Hypersil GOLD C18 column (1.9 μm, 2.1 mm × 100 mm, Thermo scientific). The column temperature was 25 °C. The mobile phase consisted of the solvent A (acetonitrile) and B (0.1 % formic acid). The gradient elutionstarted at 30 % A, ramped linearly to 50 % A in 15 min, then directly increased to 100 % A for 0.5 min and returned to the initial percentage. The flow rate was 400 μL/min and the injection volume was 5 μL. The ESI-MS was operated on negative mode at a spray voltage of 3.0 kV. Nitrogen was used as the vacuum gas, desolvation gas (1000 L/h) and cone gas (150 L/h). Argon was used as collision gas (0.15 mL/min). The temperature of the source and desolvation were set at 150 and 500 °C. Multiple reaction monitoring (MRM) was employed for quantification. The parent-to-product ion pair, cone voltage and collision energy for saikosaponin a, b_2_, c, d were described in Table 1.

**Table 1:** Mass spectrum properties of four compounds

Compound Name	Parent Ion	Product Ion	Cone Voltage (V)	Collision Voltage (V)
Saikosaponin a	779.6	617.5	100	34
Saikosaponin b_2_	779.6	617.5	100	30
Saikosaponin c	925.7	101.1	100	50
Saikosaponin d	779.7	617.5	100	36

### Validation of the quantitative analysis

The linearity calibration curves of saikosaponin a, b_2_, c, d were constructed by eight different concentrations and each concentration was analyzed in triplicate, then the calibration curves were established by the plotting peak areas versus the concentrations of standard solutions. The intra-day precision was performed by analysis of six replicates within 1 day, and the inter-day precision was determined by repeated analysis of the sample in consecutive 3 days. Recovery was determined using the spiked samples. Known amount of standard solution was added into accurately weighed Radix Bupleuri samples. The mixtures were extracted and analyzed using the method described in sample preparation for evaluating the accuracy. The average recoveries were determined by the following equation: Recovery (%) = (Observed amount-Original amount)/Spiked amount × 100 %. For repeatability test, six independent sample solutions were prepared and analyzed. Variations were expressed by relative standard deviation (RSD) in above three tests.

### Statistical analysis

Data analysis was performed using SPSS 17.0, all datas were expressed as mean ± SD. One way ANOVA tests were applied when homogeneity of variance assumptions are satisfied, otherwise the equivalent non-parametric test were used. *P* < 0.05 was considered significant.

## Results

### Effect of VBRB on ALT and AST level

**Fig. 1:**
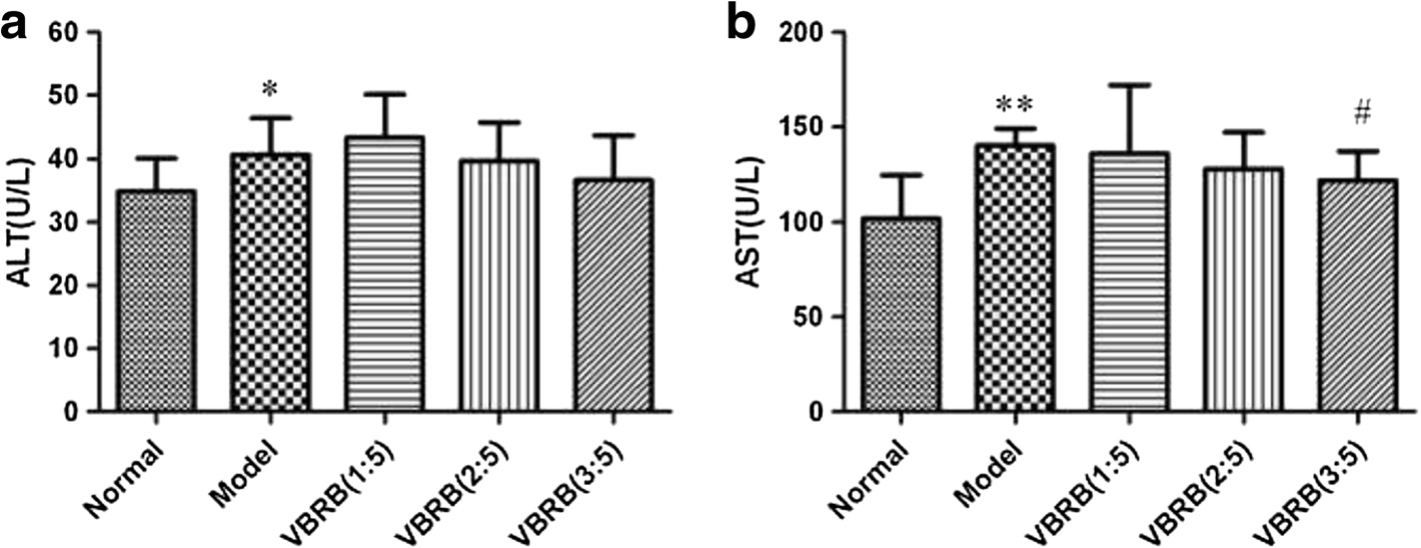
Effect of vinegar-baked Radix Bupleuri on ALT (**a**) and AST (**b**) level (* *P* < 0.05, **P < 0.01 compared with normal group; #*P* < 0.05 compared with model group)

Effects of VBRB processed by different amount of vinegar on the content of ALT and AST were shown in Fig. 1. The contents of ALT and AST in model rats were significantly higher than those in normal control group (*P* < 0.05, *P* < 0.01). After treated with VBRB (1:5), VBRB (2:5), VBRB (3:5), the ALT and AST level were decreased with the increased amount of vinegar, but not significant except for the AST level in group VBRB (3:5) rats (*P* < 0.05).

### Effect of VBRB on TBIL, DBIL and TBA level

**Fig. 2:**
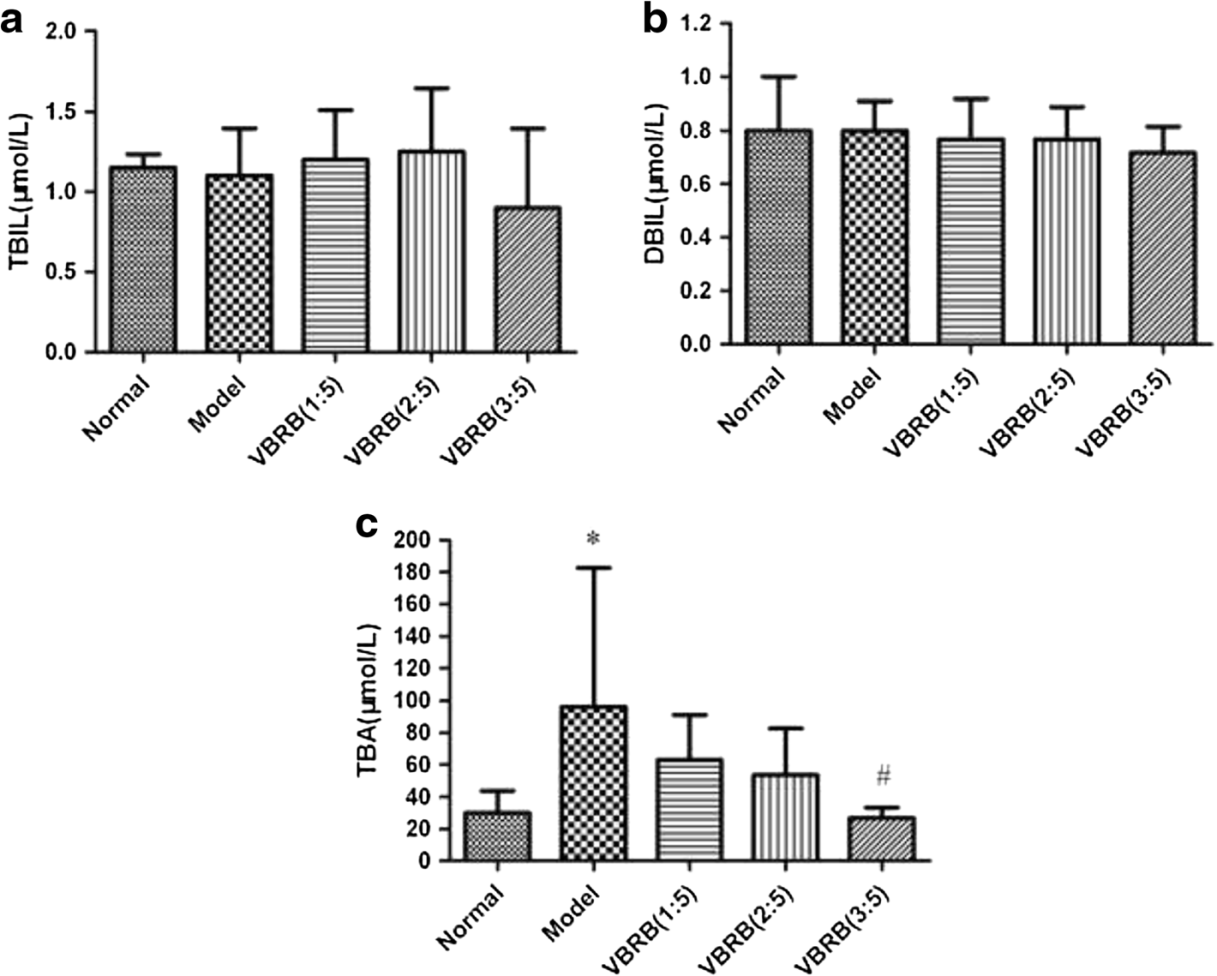
Effect of vinegar-baked Radix Bupleuri on TBIL (**a**), DBIL (**b**) and TBA (**c**) level (* *P* < 0.05 compared with normal group; #*P* < 0.05 compared with model group)

Effects of VBRB processed with different amount of vinegar on contents of TBIL, DBIL and TBA were shown in Fig. 2. TBIL and DBIL level in all group didn’t change remarkably, but contents of TBA increased significantly in model rats compared to normal control group (*P* < 0.05). After treatment, the TBA level were decreased in sequence from VBRB (1:5), VBRB (2:5) to VBRB (3:5), and only the difference in group VBRB (3:5) were significant (*P* < 0.05).

### Comparison of the extracting rates of VBRB processed with different amount of vinegar effect

The extraction rates of different extraction parts of Radix Bupleuri and VBRB (calculated by raw material) were listed on the Table 2. The results showed that the extracting rates of total extract increased as the proportion of vinegar increased, so as the part of BC1, BC2 and BC3.

**Table 2:** The extracting rates of every part of Radix Bupleuri and it’s different proportion of vinegar processing product

Samples	BC1 (%)	BC2 (%)	BC3 (%)	Total (%)
Radix Bupleuri	5.108	5.240	4.339	14.687
VBRB (1:5)	5.253	5.348	4.875	14.593
VBRB (2:5)	5.385	5.877	4.996	16.259
VBRB (3:5)	6.763	6.214	6.547	18.921

### Method validation of the quantitative analysis

**Fig. 3:**
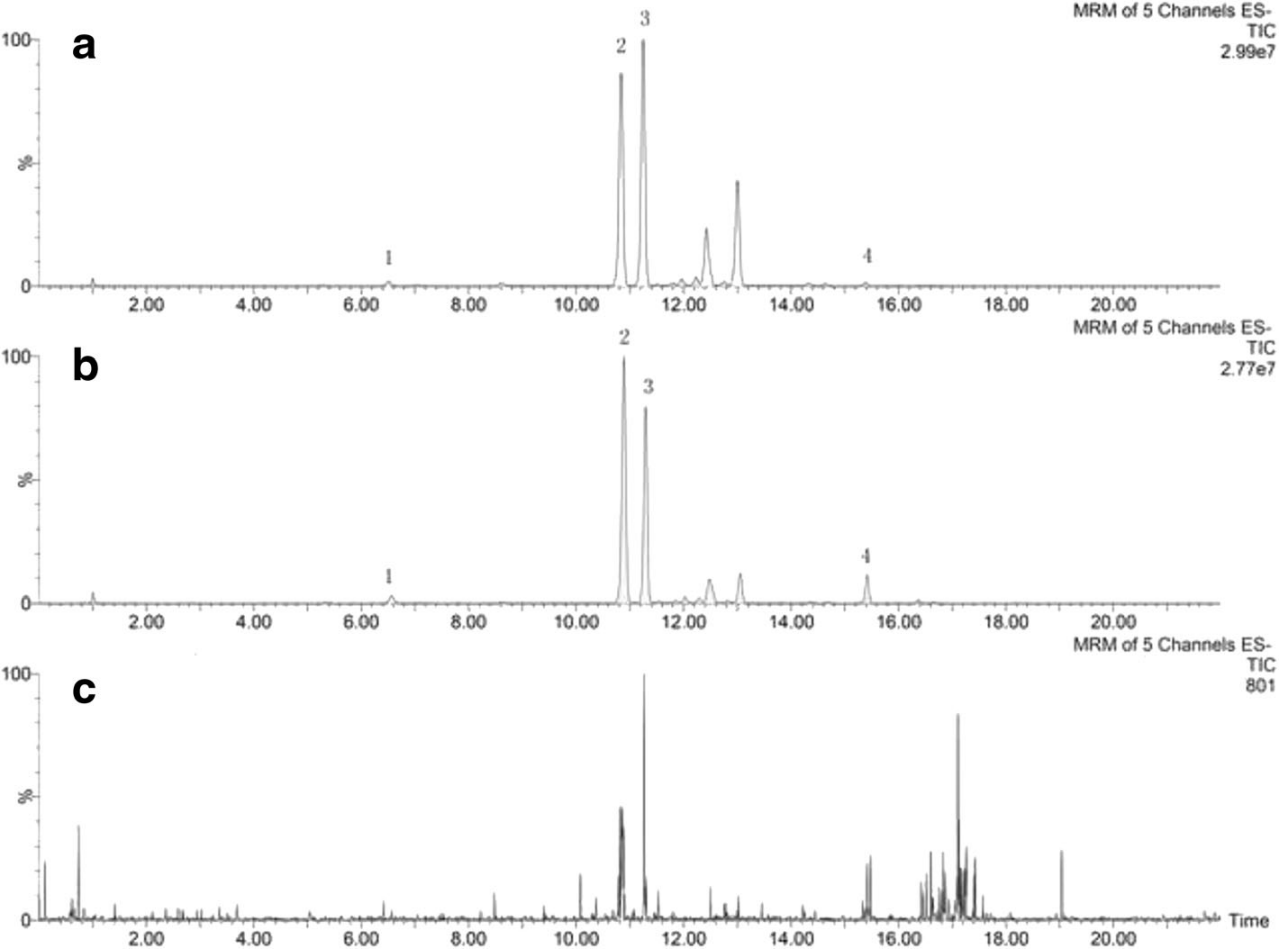
UPLC-MS/MS chromatographs of water extract of VBRB (3:5) (**a**), water extract of Radix Bupleuri (**b**) and blank solution (**c**) (*1*. saikosaponin c; *2*. Saikosaponin a; *3*. saikosaponins b_2_; *4*. saikosaponin d)

The calibration curves were prepared by assaying standard solutions as described above. The regression equations were constructed by the peak area (y) vs. concentration (x, ng/mL) and the result showed the linear relationship was good (r^2^ ≥ 0.9990). The limit of quantitation (LOQ) of saikosaponin a, b_2_, c, d were in the range of 4.03–14.55 ng/mL (Table 3). The intra- and inter-day precisions calculated as RSD were 1.42–3.89 % and 1.80–3.58 %, respectively. The overall repeatability variations were 2.84–4.08 % and the recoveries of analytes were higher than 98 %. These results indicated that this UPLC-MS/MS method is precise, accuracy and sensitive enough for simultaneously quantitative evaluation of the four saikosaponins in VBRB. Representative TIC chromatograms of samples are shown in Fig. 3.

**Table 3:** Calibration curves, linear range and LOQ of four saikosaponins

Analytes	Calibration curve	r^2^(*n* = 3)	Linear range (ng/mL)	LOQ (ng/mL)
Saikosaponin a	y = 24.935x − 141.99	0.9995	25.60–3200	6.40
Saikosaponin b_2_	y = 117.23x + 1077.3	0.9996	16.15–2020	4.03
Saikosaponin c	y = 135.96x + 200.53	0.9999	14.55–1820	7.28
Saikosaponin d	y = 103.19x − 274.17	0.9999	17.28–2160	4.32

### Quantitative determination of saikosaponins in Radix Bupleuri and VBRB

The established UPLC-MS/MS method has been successfully applied to determination of four saikosaponins in Radix Bupleuri which processed by different amount of vinegar. The retention time of saikosaponin a, b_2_, c, d under the optimized UPLC-MS assay were 6.47, 10.84, 11.22 and 15.35 min, respectively. The contents of four saikosaponins are summarized in Table 4.

**Table 4:** Contents of four saikosaponins assayed in samples of Radix Bupleuri extracted with water

Samples	Content (mg/g)			
	Saikosaponin a	Saikosaponin b_2_	Saikosaponin c	Saikosaponin d
Radix Bupleuri	11.04	4.84	3.52	0.93
VBRB (1:5)	6.44	6.95	1.56	0.16
VBRB (2:5)	4.99	7.32	2.00	0.18
VBRB (3:5)	4.75	9.33	1.98	0.13

## Discussion

Traditional Chinese processing techniques (PaoZhi) is one of the commonly used methods for changing drug property, reducing toxicity and improving effects of TCM. It was reported that VBRB had a stronger effect than Radix Bupleuri on bile secreting and hepatoprotective effects [11], and previous study also showed the pharmacological change may be related with the change of components in VBRB [17, 18]. However, the pharmacological study and the component study usually did separately, and temperature, pressure and soaking time were also affecting factors during vinegar processing technology. Therefore, the results couldn’t fully explain the link between components and pharmacology. This paper exclusively explored the influence of vinegar amount on hepatoprotective effect of VBRB and its corresponding change of components after controlling for other factors for the first time.

1:5, 2:5 and 3:5 were the ratios of vinegar to Radix Bupleuri which usually used in the literature. In our previous technology experiments, Vinegar could not be absorbed sufficiently by Radix Bupleuri when the ratio up to 4:5 and the content of saikosaponin b_2_ changed a little over the ratio of 3:5. Therefore we chose these three ratios for this study.

VBRB usually used in clinic curing liver related disease such as hypochondriac pain, hepatic adipose infiltration, hepatitis etc., which indicated its effect may mainly in liver protection. Alcohol, high lipids diet and abnormal emotion are the main cause of uninfected liver injury, and abnormal emotion especially anger induced model had the benefit of stable and at a shorter time to establish [19, 20]. Therefore we chose this model by angering rats through clamping tails. ALT and AST, are the indexes of evaluating the severity of liver damage. When liver cells are necrotic, the index of ALT and AST increased, and the extent of increased level are accordance with the severity of liver damage. TBIL, DBIL and TBA are the indexes reflecting the secretion, excretion of the bile and the ability to detoxify of liver. Among them, TBA reflects a more sensitive liver function and its level positively correlated with the degree of liver damage. Compared the index of liver function before and after model establishment, the level of ALT, AST and TBA changed obviously which was proved that rats angered by painful stimulus, and anger lead to liver dysfunction. VBRB could improve the liver injury by decreasing the index levels of liver especially the AST and TBA levels on model induced by anger. Among them, the most effective group was group VBRB (3:5).

The efficacy of medicine is closely related to their material basis, so we analyzed the change of constituents of VBRB after processed by different amount of vinegar. The extracting rates of total extract increased with the increasing amount of vinegar in the processing, and the trend was the same in extraction parts of BC1, BC2 and BC3. The result showed that the best amount of vinegar in the processing was 3:5, and this is accordance with that of the results of pharmacology experiment. BC1, mainly containing polysaccharides, had the effect of regulate and enhance immunity [21]. BC3 was mainly composed of low molecular weight water soluble components [22], no reports were found on its pharmacological effects and needed further study. BC2 was mainly composed of saponins which were also the main and bioactive components of VBRB [22–24], so we investigated the saponins to seeking the main active compounds of VBRB in follow-up experiment. The results showed that the native saponins (saikosaponin a, c, d) decreased, and the secondary saponin (saikosaponin b_2_) increased gradually with the increasing proportion of vinegar. Modern research considered that native saponins (saikosaponin a, c, d) were the main effective compounds of Radix Bupleuri, so these saponins usually used as the index components of Radix Bupleuri [25–27]. However, similar with our results, other study also showed that secondary saponins (saikosaponin b_1_, b_2_) were increased, while the saikosaponin a, saikosaponin c, and saikosaponin d were decreased after the vinegar-baking process [28, 29], and a higher saikosaponin b content also follow a better hepatoprotective effect [17], in spite of that the model they used is CCl_4_ induced liver injury. It was also reported that the saikosaponin b_2_ had strong pharmacological effects which could be an efficient inhibitor of early hepatitis C virus entry, including neutralization of virus particles, preventing viral attachment, and inhibiting viral entry/fusion [30]. In this paper, high content of saikosaponin b_2_ in water extract illustrated that only using the native saponins as the index component of Radix Bupleuri were not complete and realistic. With the increasing amount of vinegar, the secondary saponins even should be more important index compounds. Combined with pharmacological study, we speculated that saikosaponin b_2_ may be the main active components of VBRB.

## Conclusions

In the present study, we compared the hepatoprotctive effects of Radix Bupleuri after processing with different amount of vinegar (1:5; 2:5, 3:5) on anger induced liver damage rats. The group treated with VBRB (3:5) showed the best activity. Meanwhile, we investigated the changes of the components and found that the native saponins (saikosaponin a, c, d) decreased, and the secondary saponin (saikosaponin b_2_) increased significantly with the increasing proportion of vinegar. So we concluded that the best proportion of vinegar to Radix Bupleuri in vinegar-processing technology was 3:5 and saikosaponin b_2_ may be one of the key efficacy components of VBRB.

## Abbreviations

ALT: alanine aminotransferase
AST: aspertate aminotransferase
DBIL: direct bilirubin
LOQ: limit of quantitation
MRM: multiple reaction monitoring
RSD: relative standard deviation
TBA: total bile acid
TBIL: total bilirubin
TCM: traditional Chinese medicine
UPLC-MS/MS: ultra-performance liquid chromatography tandem mass spectrometry
VBRB: vinegar-baked Radix Bupleuri
